# Delivering Lifesaving Thrombolysis for Acute Myocardial Infarction in an Underserved Region of Ghana: Lessons From the First Successful Case

**DOI:** 10.7759/cureus.103036

**Published:** 2026-02-05

**Authors:** Abednego Attoh, Abdul-Subulr Yakubu, Dominic Akansiadi Akaateba, John Chukwuebuka Ota, Klein Khol Bezeng

**Affiliations:** 1 Internal Medicine and Therapeutics, Upper West Regional Hospital, Wa, GHA; 2 Cardiology, Tamale Teaching Hospital, Tamale, GHA; 3 Emergency, Upper West Regional Hospital, Wa, GHA

**Keywords:** low- to middle-income countries, myocardial infarction, northern ghana, pci, pci capable, reperfusion, revascularization, rural ghana, st elevation myocardial infarction, thrombolysis

## Abstract

The management of acute coronary syndrome in northern Ghana is constrained. This is because no facility offers percutaneous coronary intervention. Thrombolysis is the only feasible reperfusion option, yet it remains significantly underutilized due to numerous barriers. In this case report, we describe the management of a 53-year-old man with ST-elevation myocardial infarction, highlighting the routine challenges faced in treating such patients in northern Ghana and identifying opportunities for improvement.

## Introduction

Cardiovascular diseases are on the ascendancy in low- to middle-income countries like Ghana and are associated with a high mortality rate [1-3]. In 2021, ischemic heart disease, which includes myocardial infarction, accounted for an estimated 9 million deaths globally [4]. Early reperfusion therapy, preferably percutaneous coronary intervention (PCI), is crucial for reducing mortality in patients with ST-elevation myocardial infarction (STEMI), as it achieves higher reperfusion rates than thrombolysis [5,6]. However, thrombolysis remains a viable alternative, particularly in resource-limited settings, achieving high rates of recanalization and reducing mortality by up to 25% [7-9]. The most significant benefit has been observed in patients with anterior STEMI [9].

In Sub-Saharan Africa, thrombolysis remains the most frequently reported and readily accessible revascularization method, although its utilization varies widely [10]. Reperfusion therapy remains largely inaccessible to most Ghanaians. In the absence of PCI in northern Ghana, thrombolytic therapy is currently offered only at the region’s largest tertiary referral center, the Tamale Teaching Hospital (TTH) [11]. Over a one-year period, only seven of 42 patients with acute STEMI presenting to TTH underwent thrombolysis, with an in-hospital mortality of 14.3% compared to 25.7% among those who received no reperfusion therapy [12].

Lack of specialist care, inadequate health infrastructure, weak referral systems, and systemic delays contribute to the underutilization of thrombolysis outside tertiary referral facilities in northern Ghana [10-12]. The Upper West Region, one of Ghana's five northern regions, has the highest proportion of rural residents, according to the 2010 national population census [13]. The Upper West Regional Hospital (UWRH), the region's main referral facility, was largely without specialist physician services until 2021 [14]. We present a case of acute STEMI at UWRH managed with systemic thrombolysis, highlighting the challenges encountered, lessons learned, and opportunities for improvement.

## Case presentation

**Figure 1:**
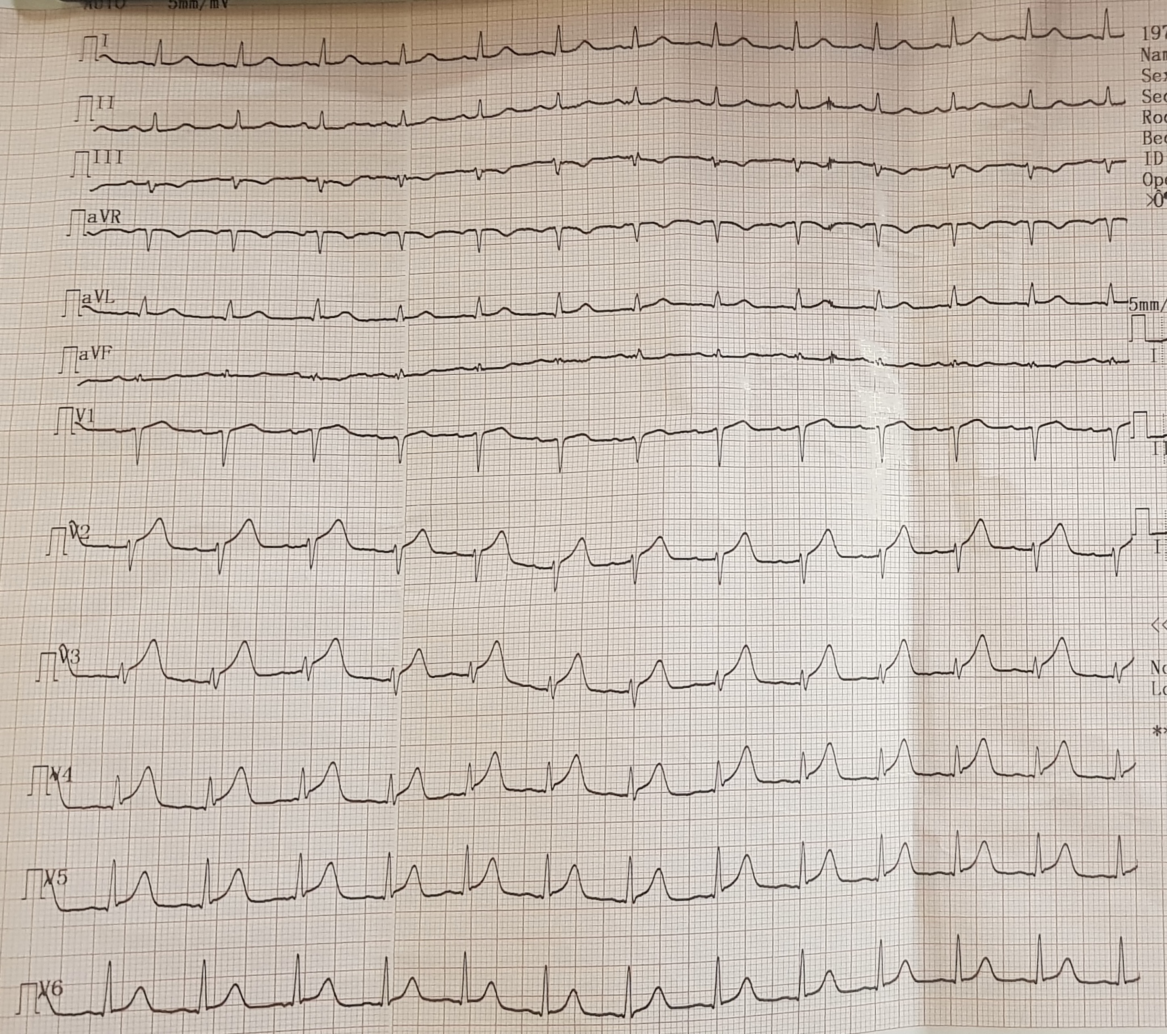
Initial ECG showing hyperacute T-waves seen in V2-V6 (voltage - 5 mm/mV; speed 25 mm/sec) ECG: electrocardiogram

The patient was a 53-year-old man who was diagnosed with hypertension a year prior to presentation but was not compliant with his medication. He presented with a three-hour history of severe central chest pain associated with restlessness and diaphoresis. He denied any history of dyspnea, orthopnea, or paroxysmal nocturnal dyspnea. He was a non-smoker and did not use illicit drugs. An electrocardiogram (ECG) done at a peripheral facility before arrival showed sinus tachycardia with hyperacute T-waves in leads V2-V6 (Figure 1).

**Figure 2:**
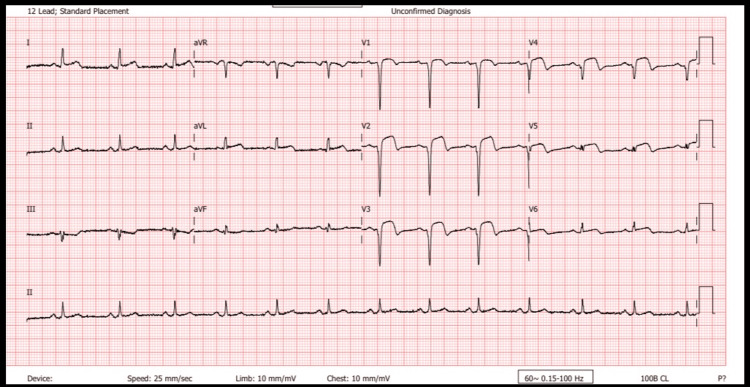
ECG showing deep Q-waves in leads V1-V4 with ST elevations in V1-V5 ECG: electrocardiogram

He was restless and in severe pain. He was also anicteric and afebrile, with no pallor or peripheral edema. His blood pressure was 146/96 mmHg, heart rate 82 bpm, and oxygen saturation 99% on room air. Heart sounds were normal, and the chest was clear to auscultation. Abdominal examination revealed central adiposity; the patient weighed 91 kg. The rest of his examination was unremarkable. Random blood glucose at the time was 7.2 mmol/L. A 12-lead ECG done on arrival showed ST elevations in leads V1-V5 with pathological Q-waves now present in V1-V4, findings consistent with an acute anteroseptal STEMI (Figure 2).

Interventions

**Figure 3:**
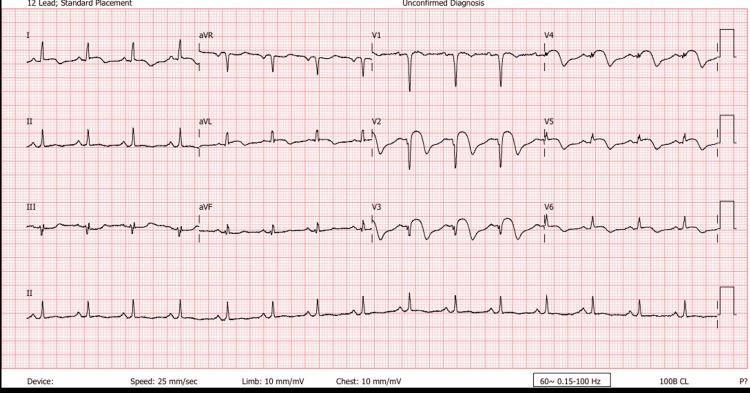
ECG done six hours post thrombolysis showing anteroseptal Q-waves with resolution of ST elevations and development of T-wave inversions ECG: electrocardiogram

**Figure 4:**
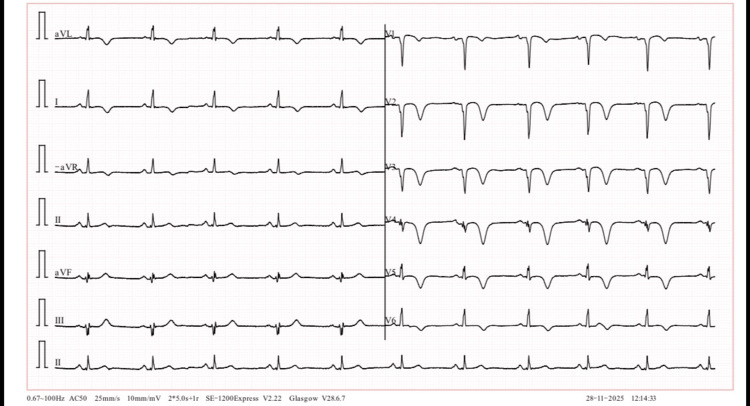
ECG showing pathological Q-waves with persistent T-wave inversions at one-month post thrombolysis ECG: electrocardiogram

The nearest PCI-capable facility was more than 120 minutes away, so the patient was immediately counseled on thrombolytic therapy as a means of reperfusion, after excluding any contraindication. The time interval from arrival to thrombolytic agent administration (door-to-needle time) was approximately 60 minutes. Thrombolysis was performed with 100 mg of alteplase administered as an infusion in accordance with standard protocols. He remained stable throughout the thrombolysis period. His vital signs after the procedure were normal, and chest pain resolved within 45 minutes of thrombolysis. His serum cardiac troponin levels were markedly elevated (Table 1). He received the following medications: oral aspirin 75 mg daily (after a loading dose of 300 mg), oral clopidogrel 75 mg daily (after a loading dose of 300 mg), oral atorvastatin 80 mg daily, oral carvedilol 3.125 mg 12 hourly, and subcutaneous enoxaparin 80 mg 12 hourly whilst on admission. Sublingual glyceryl nitrate was administered as necessary for chest pain. Serial 12-lead ECGs showed pathologic Q-waves, resolution of ST elevations, and the appearance of inverted T-waves (Figures 3-4).

An echocardiogram showed an akinetic anteroseptal wall in the mid-to-apical regions of the left ventricle and a left ventricular ejection fraction of 56%. He had grade II diastolic dysfunction and mild mitral regurgitation. No intracardiac thrombi were found. He was discharged home three days after admission and remained stable and pain-free at his eight-day and one-month reviews. His medication was updated to include lisinopril 5 mg daily and empagliflozin 10 mg daily. The plan is to continue dual antiplatelet therapy for one year. He has been scheduled for an updated echocardiogram in three to six months.

**Table 1:** Summary of laboratory results eGFR: estimated glomerular filtration rate, hs: high-sensitivity, HDL: high-density lipoprotein, LDL: low-density lipoprotein, WBC: white blood cell count, Hb: hemoglobin, Plt: platelet

Parameter	Value	Reference
Urea	3.88 mmol/l	3.2-7.3 mmol/l
Creatinine	111.67 umol/l	53-97 umol/l
eGFR	68 ml/min/1.73 m2	-
Sodium	139.10 mmol/l	135-145 mmol/l
Potassium	3.41 mmol/l	3.5-5.5 mmol/l
Chloride	100.30 mmol/l	100.30 mmol/
Hs troponins	128.9 ng/l	0-14 ng/l
Total cholesterol	4.47 mmol/	3.6-5.2 mmol/l
HDL-cholesterol	0.60 mmol/l	1.1-3.2 mmol/l
Triglycerides	1.34 mmol/l	0.40-1.54 mmol/l
LDL-cholesterol	3.26 mmol/l	0.00-3.11 mmol/l
WBC	6.76 x10^3/ul	4 -7.5x10^3/ul
Hb	14 g/dl	13.5-18.0g/dl
Plt	161 x10^3/ul	150-400 x10^3/ul

## Discussion

This case highlights the feasibility of thrombolytic therapy for acute STEMI in resource-limited rural Ghana, where immediate access to primary PCI is unavailable even when patients present early. Notably, this patient was managed by a physician under the remote guidance of an off‑site cardiologist via telemedicine, thereby demonstrating the feasibility of this approach.

Current guidelines recommend that, in non-PCI-capable facilities, the door-to-needle time be less than 30 minutes [15-17]. In this first documented case of thrombolysis in a facility (UWRH), reperfusion was achieved in approximately 60 minutes. With appropriate systems and infrastructure in place, it may be possible to consistently meet the recommended timelines, thereby maximizing therapeutic benefit and potentially saving up to 65 lives per 1000 patients treated [9,17]. Early symptom relief, as achieved in this case, together with sustained clinical stability, is a well‑recognized indicator of successful thrombolysis and is strongly associated with improved prognosis, particularly when reperfusion occurs prior to extensive myocardial necrosis [18,19]. However, the patient’s ECG demonstrated persistent Q-waves, and his echocardiogram revealed wall motion abnormalities, consistent with residual loss of viable myocardium. Initiation of thrombolysis at the time of his initial ECG might have yielded a more favorable outcome.

In many low- to middle-income countries, timely reperfusion remains challenging due to limited infrastructure, delayed presentation, scarcity of specialist services, and cost of medications [11,12]. Thrombolytic medications are not reimbursed by Ghana's National Health Insurance Scheme (NHIS), creating a significant financial burden for most patients. A deliberate policy to ensure the continuous availability and affordability of lifesaving medications, including thrombolytics, should be instituted, with coverage under the NHIS. Adapting context‑appropriate guidelines and protocols for the management of cardiac emergencies, including acute coronary syndromes, would facilitate effective implementation while remaining sensitive to local realities and constraints [20].

Basic ECG training for frontline clinicians, supplemented by telecardiology, streamlined referral pathways, and comprehensive emergency response systems, would enhance the detection and management of life-threatening cardiac conditions. Collectively, these interventions have the potential to improve cardiovascular outcomes at a population level.

In this case, the presence of an on‑site physician specialist, access to a cardiologist via telephone, and the availability of alteplase within the hospital enabled the timely delivery of lifesaving care despite significant resource limitations.

## Conclusions

In the absence of a PCI-capable facility across northern Ghana, establishing a robust thrombolysis program for eligible patients remains paramount. Prompt recognition and early initiation of therapy are critical determinants of successful thrombolysis. To further reduce treatment delays and broaden access to timely reperfusion, system‑level interventions would be required. This case underscores both the safety and feasibility of thrombolysis in a resource‑constrained setting, even without on‑site cardiology support.
